# A metal artifact reduction method for a dental CT based on adaptive local thresholding and prior image generation

**DOI:** 10.1186/s12938-016-0240-8

**Published:** 2016-11-04

**Authors:** Mohamed A. A. Hegazy, Min Hyoung Cho, Soo Yeol Lee

**Affiliations:** Department of Biomedical Engineering, Kyung Hee University, Yongin-Si, Gyeonggi-do 446-701 South Korea

**Keywords:** Metal artifact reduction, Dental CT, Adaptive local thresholding, Prior image, Iterative image reconstruction

## Abstract

**Background:**

Metal artifacts appearing as streaks and shadows often compromise readability of computed tomography (CT) images. Particularly in a dental CT in which high resolution imaging is crucial for precise preparation of dental implants or orthodontic devices, reduction of metal artifacts is very important. However, metal artifact reduction algorithms developed for a general medical CT may not work well in a dental CT since teeth themselves also have high attenuation coefficients.

**Methods:**

To reduce metal artifacts in dental CT images, we made prior images by weighted summation of two images: one, a streak-reduced image reconstructed from the metal-region-modified projection data, and the other a metal-free image reconstructed from the original projection data followed by metal region deletion. To make the streak-reduced image, we precisely segmented the metal region based on adaptive local thresholding, and then, we modified the metal region on the projection data using linear interpolation. We made forward projection of the prior image to make the prior projection data. We replaced the pixel values at the metal region in the original projection data with the ones taken from the prior projection data, and then, we finally reconstructed images from the replaced projection data. To validate the proposed method, we made computational simulations and also we made experiments on teeth phantoms using a micro-CT. We compared the results with the ones obtained by the fusion prior-based metal artifact reduction (FP-MAR) method.

**Results:**

In the simulation studies using a bilateral prostheses phantom and a dental phantom, the proposed method showed a performance similar to the FP-MAR method in terms of the edge profile and the structural similarity index when an optimal global threshold was chosen for the FP-MAR method. In the imaging studies of teeth phantoms, the proposed method showed a better performance than the FP-MAR method in reducing the streak artifacts without introducing any contrast anomaly.

**Conclusions:**

The simulation and experimental imaging studies suggest that the proposed method can be used for reducing metal artifacts in dental CT images.

## Background

Metal artifacts often compromise readability of computed tomography (CT) images when the patient has metal implants or metal devices in his or her body. Metal artifacts usually appear as strong streaks around the metallic objects because the attenuation coefficients of metallic objects are much higher than those of human body tissues. High attenuation of the x-ray beam in the metallic objects induces signal saturation in the projection data, beam hardening, photon scattering, and photon starvation, all of which contribute to producing metal artifacts.

Many metal artifact reduction (MAR) methods have been developed since the introduction of a medical CT to the clinical field decades ago. Most of MAR techniques identify the trace of metallic objects on the projection data and then modify the projection data at the metal trace by interpolating the pixel values around the metal trace [1–6]. The interpolation process is computationally efficient, but it often makes other artifacts in the resulting images due to the interpolation errors [7]. To reduce the interpolation errors, many forward-projection-based methods have been introduced [8–13]. In the forward-projection-based methods, the missing data at the metal trace are inferred by forward-projecting the prior images. To reduce the metal artifacts effectively, generating prior images, that can provide missing projection data at the metal trace, is critical. In generating prior images, precise metal segmentation of the CT images is essential. Any wrong metal segmentation would result in residual metal artifacts after the metal artifact correction. Particularly in a dental CT, wrong metal segmentation is a big concern since teeth have x-ray attenuation coefficients that are not very different from those of metallic objects [14]. Therefore, segmentation of metal regions in dental CT images is often unsatisfactory since teeth are mistakenly identified as metallic objects. To further reduce metal artifacts, iterative image reconstruction methods, such as the expectation maximization method or the algebraic reconstruction technique (ART), can be used with some regularization [15–19]. Total variation minimization is often used for regularization in the iterative image reconstruction [20, 21]. However, the iterative image reconstruction with regularization is computationally expensive. Some hybrid methods have been also introduced to balance the metal artifact reduction and the computational cost [22, 23]. Recently, a metal artifact reduction method has been introduced with consideration of the beam hardening effect of a polychromatic x-ray beam [24].

We propose a MAR technique for a dental CT in which a metal trace is identified directly from the projection data rather than from the CT images. To accurately identify a metal trace, we use the local statistics of pixel values inside and around the metal trace that has been first identified by applying global thresholding to the projection data. After identifying the metal trace from the projection data, we replace the pixel values at the metal trace with the ones computed from the prior images. For the prior image generation, we use the recently introduced method, so called fusion prior-based MAR (FP-MAR) [12]. To validate the proposed method, we have performed imaging studies of dental phantoms using a micro-CT as well as computational simulation studies. We present the simulation and experimental results with comparison to the results obtained by FP-MAR.

## Methods

### The metal artifact reduction algorithm

#### Segmentation of metal trace in the projection images

Due to higher attenuation coefficients of metallic dental devices than those of biological tissues and teeth, metallic devices make high contrast in every projection image acquired by a 3D dental CT. Once the metal regions in the projection images have been identified by exploiting the high contrast, the pixel values at the metal trace in the sinogram should be modified before the back-projection to reduce metal artifacts in the CT images. Whatever methods are employed to modify the pixel values at the metal trace in the sinogram, accurate segmentation of metal regions in the projection image is crucial for successful reduction of metal artifacts. Despite the high attenuation coefficients of metallic dental devices, segmentation of metal regions is often unsatisfactory since many objects are overlapped in the projection image. Global thresholding by applying a single threshold all over the image region may result in a smaller or bigger segmented region than the original size.

In most MAR algorithms, global thresholding is used to identify metal regions due to its computational efficiency [12]. However, small errors in the metal segmentation in the projection image would lead to residual streak artifacts in the reconstructed images. So, we expand the segmented region, obtained by the global thresholding with a little low threshold level, to the exact size. In the expansion algorithm, the result of global thresholding, $M(s,t,\theta )$, on the original projection image, $P_{orig} (s,t,\theta )$, is used as a seed for expansion. In $M(s,t,\theta )$ and $P_{orig} (s,t,\theta )$, *s* and *t* represent the horizontal and vertical axes on the detector plane, respectively, and *θ* represents the scan angle of the cone-beam-based dental CT. The global thresholding with a global threshold value $T$ is applied to the projection image set all over the scan angle:

1 $$ M(s,t,\theta ) = \left\{ {\begin{array}{*{20}c} {0\quad if \, P_{orig} (s,t,\theta ) \ge T} \\ {1\quad if \, P_{orig} (s,t,\theta ) < T} \\ \end{array} } \right. $$

in which the global threshold *T* is empirically chosen in a way that only the metallic objects are segmented. In the mask $M(s,t,\theta )$, a segmented region tends to be a little smaller than the actual size of the metallic object. In every mask $M(s,t,\theta )$, we identify the islands of zeroes in (*s,t*) domain, i.e., the metal regions. After identifying the islands of zeros, we compute the standard deviation of pixel intensity σ(*i*,*θ*), the maximum pixel intensity *A*(*i*,*θ*), and the minimum pixel intensity *B*(*i*,*θ*) at the *i*-th metal region in the projection image at the scan angle of *θ*. After that, we find the starting point, $s_{1}$, and the ending point, $s_{2}$, in every row of the island of zeroes. We define the search window *W* in which we find the exact boundary of the metallic object. The criterion to decide whether a pixel in a row within *W* actually belongs to the metal region or not is given below:

2 $$ \tilde{M}(s,t,\theta ) = \left\{ \begin{array}{ll} 0 & \quad if \, B(i,\theta ) + a\sigma (i,\theta ) \le P_{orig} (s,t,\theta ) \le A(i,\theta ) - a\sigma (i,\theta )  \\ 
& \qquad \qquad \qquad \qquad \qquad \qquad \qquad s \in [s_{1} - W,s_{2} + W] \\
1 & \quad otherwise \end{array}  \right.$$

in which a scaling factor *a* is to be found empirically to enhance the expansion performance. Since Eq. (2) takes account of the local statistics of pixel intensity inside a metal region identified by Eq. (1), the metal mask obtained by Eq. (2) better represents the actual metal region. If the metal region is overlapped with other high density structures, the maximum and minimum pixel intensities will rise so that the local lower bound and higher bound will be increased to keep the segmented region from growing too much. Since the metal region searching after the global thresholding is performed only within the small window of 2*W* around the metal boundary, the computational burden is minimal. In all the simulations and experiments in this work, we have used the scaling factor of 2. After expanding the segmented region in the horizontal direction, we apply the same rule in the vertical direction too.

**Fig. 1:**
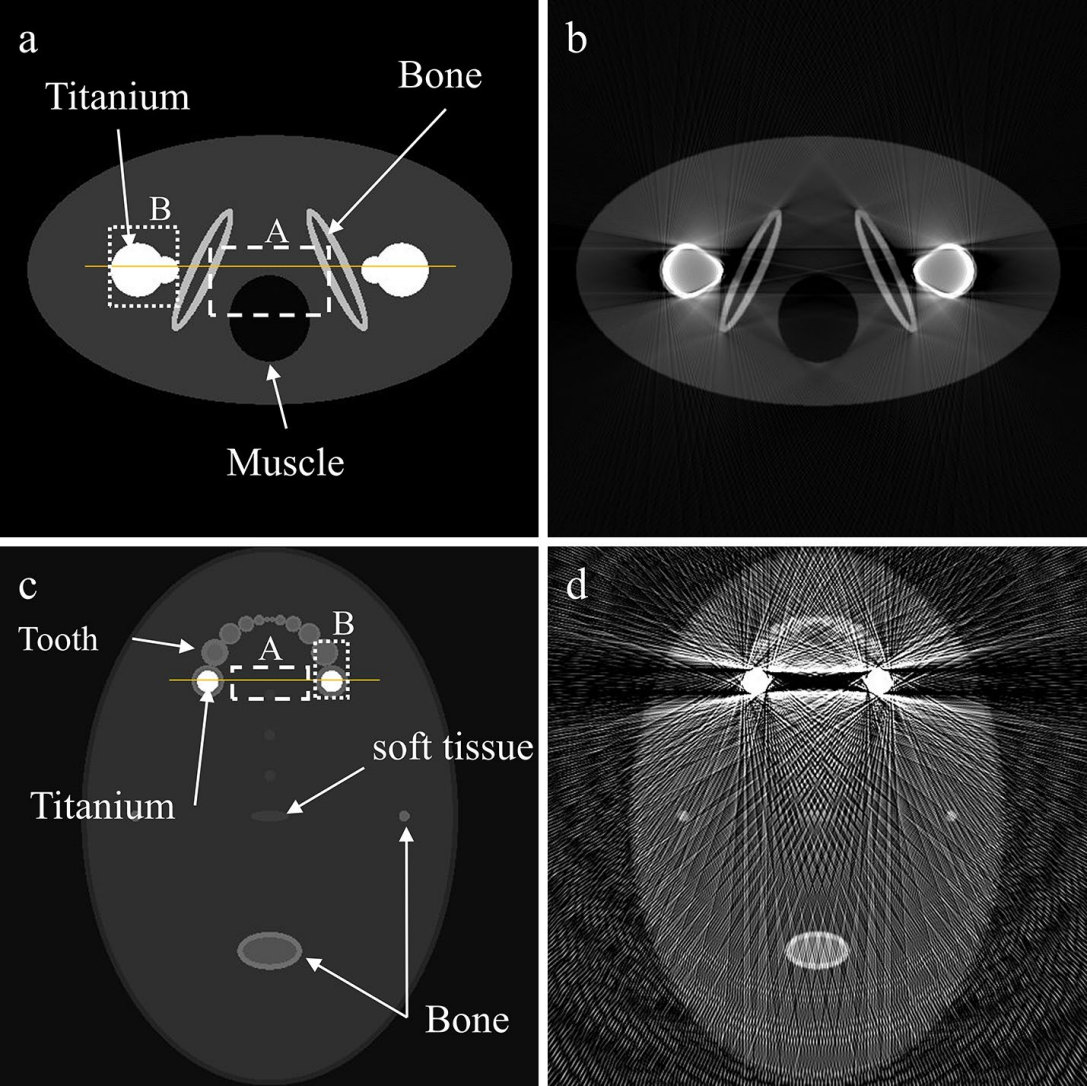
**a** The bilateral prostheses phantom that has two metallic objects in it. **b** The image of the bilateral prostheses phantom reconstructed by filtered backprojection without any metal artifact correction. **c** The dental phantom that has two metallic objects in it. **d** The image of the dental phantom reconstructed by filtered backprojection without any metal artifact correction. The ROI *A* and *B* in **a** and **b** are the regions for the evaluation of metal artifact correction

**Fig. 2:**
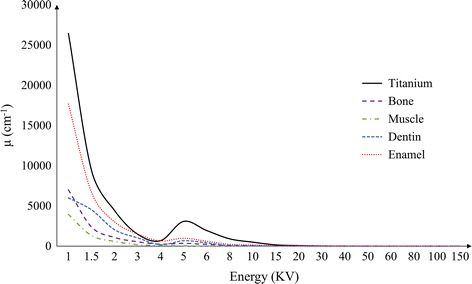
The energy dependency of the attenuation coefficients of the phantom components indicated in Fig. 1

Figure 1a, c show the 2D phantoms to be used for the computational simulation of the metal artifact generation and correction. Figure 1a simulates bilateral prostheses in the pelvis region with Fig. 1c simulating dental implants and teeth. The phantom, shown in Fig. 1a, consists of a muscle tissue, bones, and titanium implants. The phantom, shown in Fig. 1c, consists of soft tissues, bones, teeth, and titanium implants. A tooth in the phantom consists of dentin surrounded by a thin enamel layer. Figure 1b, d show the images reconstructed by the filtered backprojection without any metal artifact correction. In the image reconstruction, the number of views was 720, and the image matrix size was 512 × 512. For the image reconstruction, the projection data were computed by taking a line integral of the phantom with consideration of the energy dependency of the attenuation coefficients of the constituent parts of the phantom. Figure 2 shows the energy-dependent attenuation coefficients of the titanium, bone, enamel, dentin and muscle. The energy dependency of the soft tissue, not shown in Fig. 2, is very similar to the one of the muscle.

**Fig. 3:**
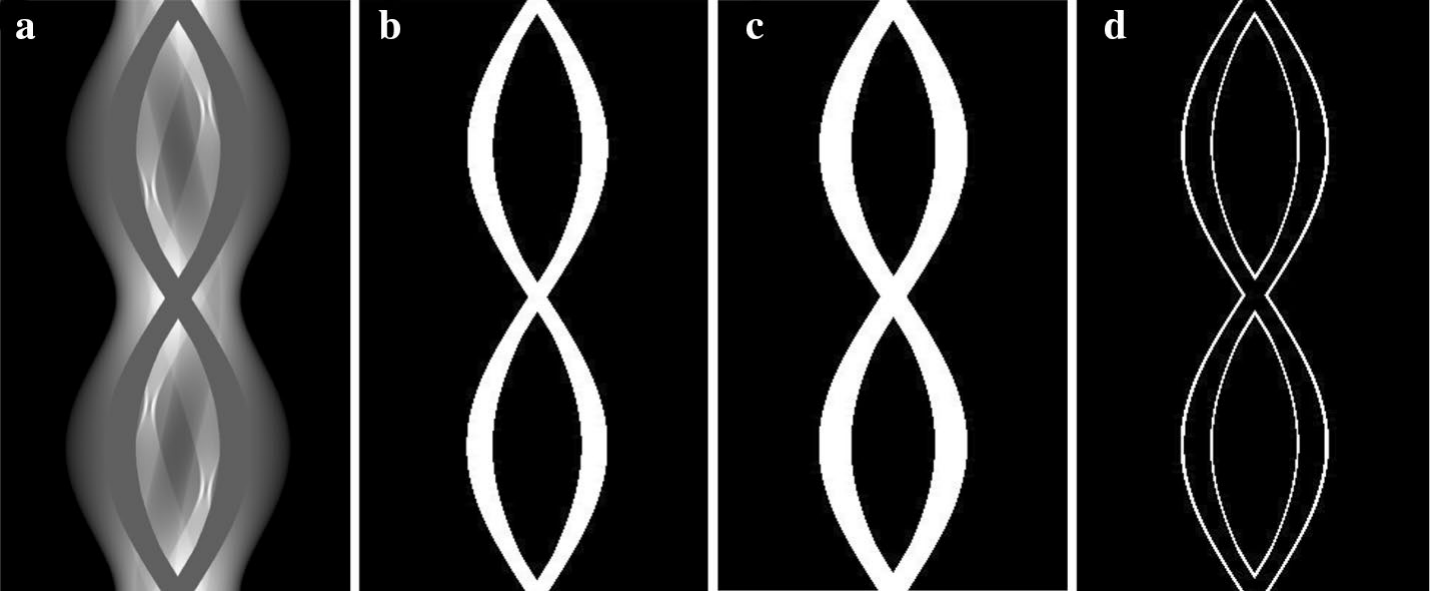
**a** The original sinogram of the phantom shown in Fig. 1a. **b** The metal mask obtained by the global thresholding. **c** The metal mask after the expansion in the horizontal direction (*W* = 30). **d** The difference between **b** and **c**

Figure 3 shows the sinograms of the simulation phantom shown in Fig. 1a during the segmentation steps. In this simulation, the phantom is two-dimensional, hence, the *t*-direction is not considered in the segmentation steps. Figure 3a shows the original sinogram of the phantom. Figure 3b shows the metal mask obtained by the global thresholding with Fig. 3c showing the metal mask after the expansion in the *s*-direction. Figure 3d shows the difference between Fig. 3b, c.

#### Prior image generation

**Fig. 4:**
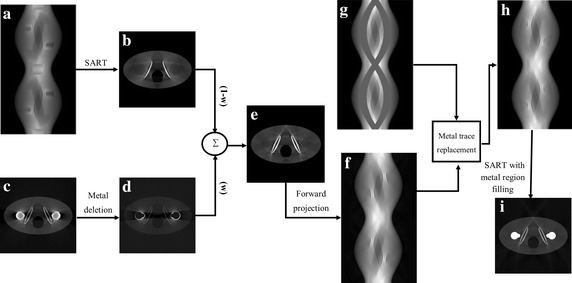
The flow chart of the proposed metal artifact reduction method. **a**, **b** The streak-free image generation, **c**, **d** the metal-free image generation, **e** the prior image generated by fusion of the two images, **f** the prior sinogram generated by the forward projection of the prior image, **g** the identified metal trace in the sinogram, **h** the corrected sinogram, **i** the final reconstructed image

After segmenting the metal parts, we compute prior images that will be used to replace the metal region in the projection image. After identifying the metal region in the projection image as described in the previous section, the projection image is multiplied by the metal mask so that the pixel values in the metal region are set to zero. Then, the pixel values in the metal region are filled with the ones computed by linear interpolation of the pixel values at both sides of a row in the metal region. Figure 4a shows the sinogram after replacing the metal region by linear interpolation row by row. From the sinogram after the linear interpolation, a streak-free image $I_{sf} \left( {x,y} \right)$ is reconstructed as shown in Fig. 4b. This will be the first component of the prior image $I_{p} \left( {x,y} \right)$. In computing the streak-free image, iterative image reconstruction methods like the simultaneous algebraic reconstruction technique (SART) are preferred since iterative image reconstructions generate less streak artifacts than filtered backprojection.

The second component of the prior image is generated from the image reconstructed from the original projection data without artifact correction, as shown in Fig. 4c. From the image shown in Fig. 4c, the metal parts are removed to get a metal-free image $I_{mf} \left( {x,y} \right)$ as shown in Fig. 4d. In the metal free image, the metal parts are nullified after identifying the metal parts by global thresholding.

The prior image is calculated by weighted summation of $I_{sf} \left( {x,y} \right)$ and $I_{mf} \left( {x,y} \right)$ as demonstrated in the Wang’s work [12]. For the weighted summation, the difference between the streak-free image and the metal-free image is computed by *D*(*x*,*y*) = *I*_*sf*_(*x*,*y*) − *I*_*mf*_(*x*,*y*). After finding the maximum value (*D*_max_) and the minimum value (*D*_min_) of *D*(*x*,*y*) over the whole image region, *D*(*x*,*y*) is normalized by:

3 $$ D_{n} (x,y) = \frac{{D(x,y) - D_{^\text{min} } }}{{D_{^\text{max} } - D_{^\text{min} } }} . $$

Then, the weighting function is computed by [12]:

4 $$ w(x,y) = \frac{1}{{1 + \left[ {\frac{{D_{n} (x,y)}}{c}} \right]^{p} }} $$

in which *p* and *c* are control parameters. The prior image is then generated by:

5 $$ I_{p} (x,y) = w(x,y)I_{mf} (x,y) + (1 - w(x,y))I_{sf} (x,y). $$

The control parameters in the weighting function, *p* and *c*, should be chosen so as to ensure both edge preservation and artifact reduction in the prior image. A small *p* can lead to residual streak artifacts, a small *c* can generate a prior image very similar to the streak-free image while a large *c* can generate a prior image very similar to the metal-free image [12]. By the trial and error approach, we have found that *p* should be between 10 and 20, *c* should be around 0.1 for small metallic objects and around 0.45 for large metallic objects.

#### Projection data correction

After computing the prior image shown in Fig. 4e, the original projection data are corrected to reduce streak artifacts. The prior projection image, *P*_prior_ (*s*,*t*,*θ*) is computed by forward-projecting the prior image as shown in Fig. 4f. The prior image has less streak artifacts, lower pixel intensity in the metal region, and less beam hardening artifacts, that is, it is similar to the original image except at the metal region. The metal region in the original projection image, shown in Fig. 4g, is replaced with the one in the prior projection image as shown in Fig. 4h:

6 $$ \overset{\lower0.5em\hbox{$\smash{\scriptscriptstyle\frown}$}}{P} (s,t,\theta ) = M(s,t,\theta )P_{orig} (s,t,\theta ) + (1 - M(s,t,\theta ))P_{prior} (s,t,\theta ) . $$

In the corrected projection image, the original projection data are kept outside the metal region. From the corrected projection data, final images are reconstructed by SART followed by metal region filling.

To quantitatively evaluate the performance of metal artifact correction, the structural similarity (SSIM) index has been used. The SSIM index was developed as a measure of structural information change from the original image to the distorted image, and it was considered to be a good measure for perceived image distortion [25]. The SSIM index was computed using the open software [26] on the regions of interest (ROIs) A and B at each simulation phantom shown in Fig. 1a, c. The ROI A is positioned in between the metal objects to evaluate the similarity hampered by the streak and shadow artifacts, and the ROI B surrounds the metallic object to evaluate the similarity of the corrected image to the original metallic structure.

### Experimental setup

**Fig. 5:**
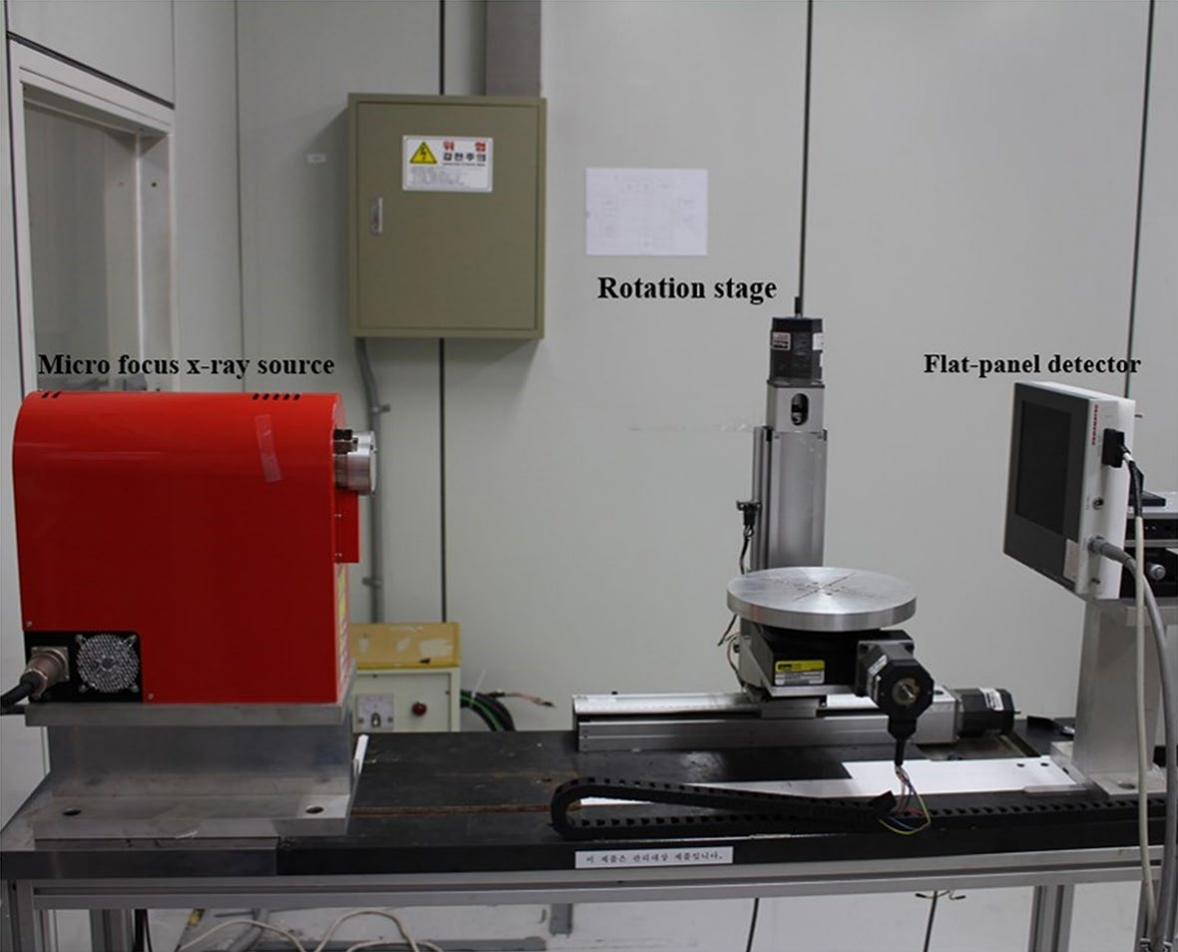
The lab-built micro-CT system consisting of a micro-focus x-ray source, a flat-panel detector and a rotating stage

We took 3D tomographic images of two dental phantoms using a lab-built micro-CT to verify the proposed MAR method. The micro-CT consists of a micro-focus x-ray tube (L8101-01, Hamamatsu, Japan) and a CsI:Tl flat panel detector (C7942, Hamamatsu, Japan). In between the micro-focus x-ray tube and the flat panel detector lies a precision rotation stage for a CT scan. The micro-focus x-ray tube has a focal spot size of 5–20 µm depending on the operating tube voltage and current. The maximum tube voltage and current are 150 kV and 500 µA, respectively. The flat panel detector has a 50 µm pixel pitch with a matrix size of 2240 × 2240. A photograph of the micro-CT system is shown in Fig. 5.

**Fig. 6:**
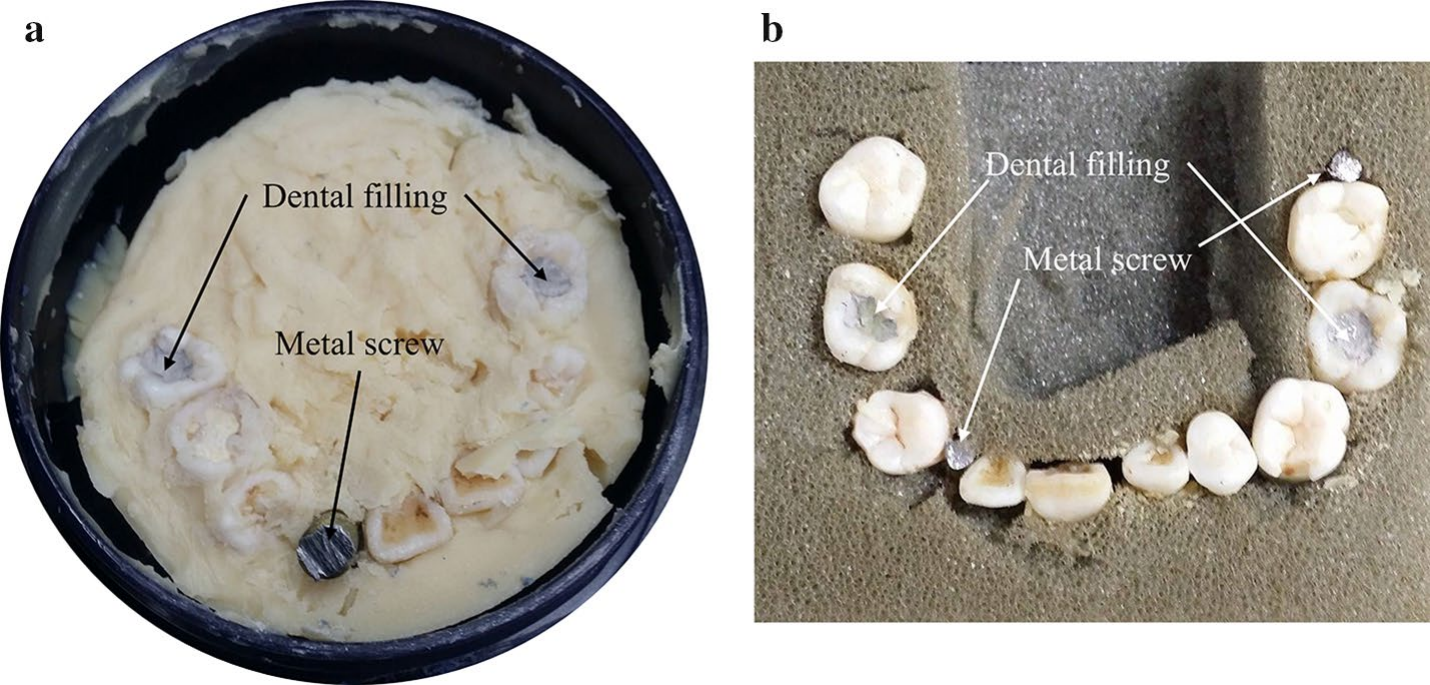
**a** The dental phantom having real human teeth, a metal screw and dental fillings. **b** The dental phantom having real human teeth, two metal screws and dental fillings

To verify the proposed MAR method experimentally, we made two dental phantom as shown in Fig. 6. The phantoms consist of real human teeth, some metal screws and amalgam dental fillings as indicated in Fig. 6. We took 3D CT images of the phantoms with a tube voltage and current of 80 kVp and 300 µA, respectively. The number of projection views was 720 over 360°, and the detector integration time was 1 ms. The source to object distance (SOD) and source to detector distance (SDD) were set to make an appropriate geometric magnification of 4.3. In the imaging experiments, the SDD and SOD were 432.5 and 331.5 mm, respectively.

## Results

**Fig. 7:**
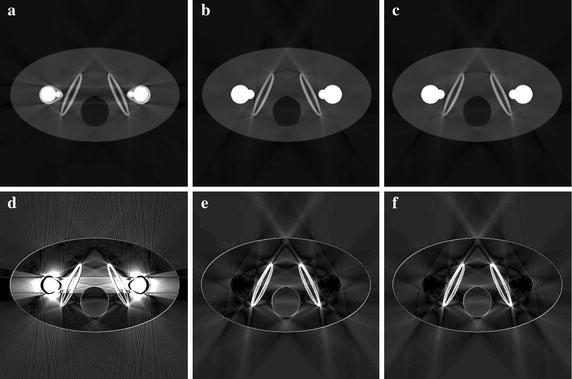
The metal artifact correction results of the bilateral prostheses phantom. **a** FP-MAR with THR = 0.05. **b** FP-MAR with THR = 0.07. **c** The proposed method with a global threshold of 0.05 (*p* = 10, *c* = 0.1). **d**–**f** Difference of **a**–**c** from the original image shown in Fig. 1a

**Fig. 8:**
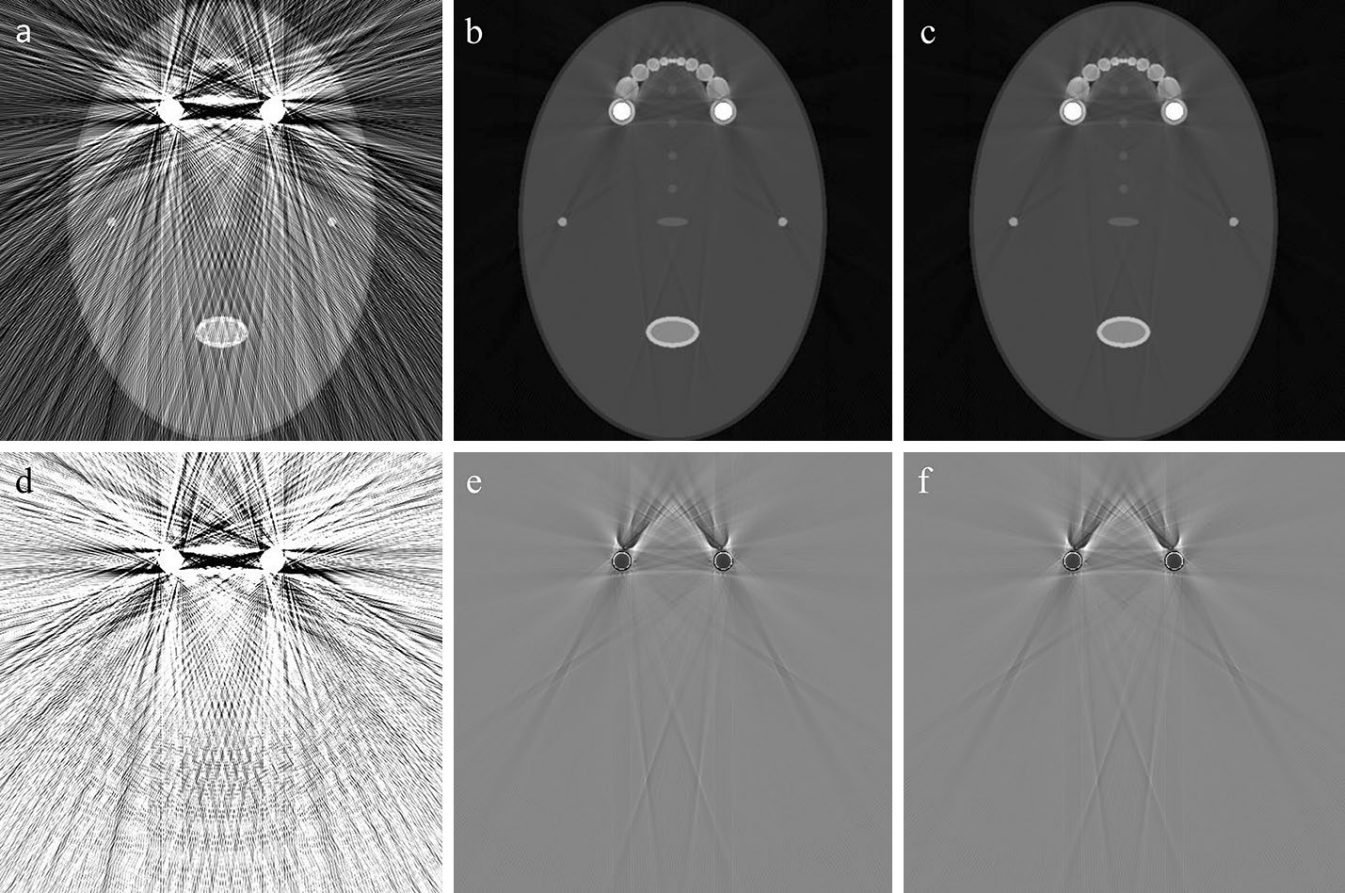
The metal artifact correction results of the dental phantom. **a** FP-MAR with THR = 0.05. **b** FP-MAR with THR = 0.07. **c** The proposed method with a global threshold of 0.05 (*p* = 10, *c* = 0.1). **d**–**f** Difference of **a**–**c** from the original image shown in Fig. 1c

We have reconstructed images from the simulated projection data of the two simulation phantoms and from the experimental projection data of the two dental phantoms using the proposed method and the FP-MAR method. Figure 7a, b show the images of the bilateral prostheses phantom reconstructed by the FP-MAR method with different global thresholds of 0.05 and 0.07, respectively. The projection data were normalized by the peak value so that they had the maximum value of 1.0 all over the scan angles. The lower global threshold results in the residual streaks and shadow artifacts as can be seen from Fig. 7a. The global threshold giving the least streak artifact has been found to be around 0.07 as can be seen in Fig. 7b. Figure 7c shows the image of the simulation phantom reconstructed by the proposed method with the global threshold of 0.05 and the weighting parameters of *p* = 10 and *c* = 0.1. Figure 7d–f show the difference images of Fig. 7a–c, respectively, from the original phantom image. Similarly, Fig. 8 shows the simulated dental phantom images reconstructed by the FP-MAR method and the proposed method along with the difference images. The lower global threshold, 0.05, for the FP-MAR method results in the residual streaks and shadow artifacts as can be seen from Fig. 8a. The optimal global threshold, 0.07, for the FP-MAR method results in the least streak artifact as can be seen in Fig. 8b. Figure 8c shows the image reconstructed by the proposed method with the global threshold of 0.05 and the weighting parameters of *p* = 10 and *c* = 0.1. In the difference images shown in Fig. 8d–f, the proposed method (Fig. 8f) also shows a performance similar to the optimal case in the FP-MAR method (Fig. 8e).

**Fig. 9:**
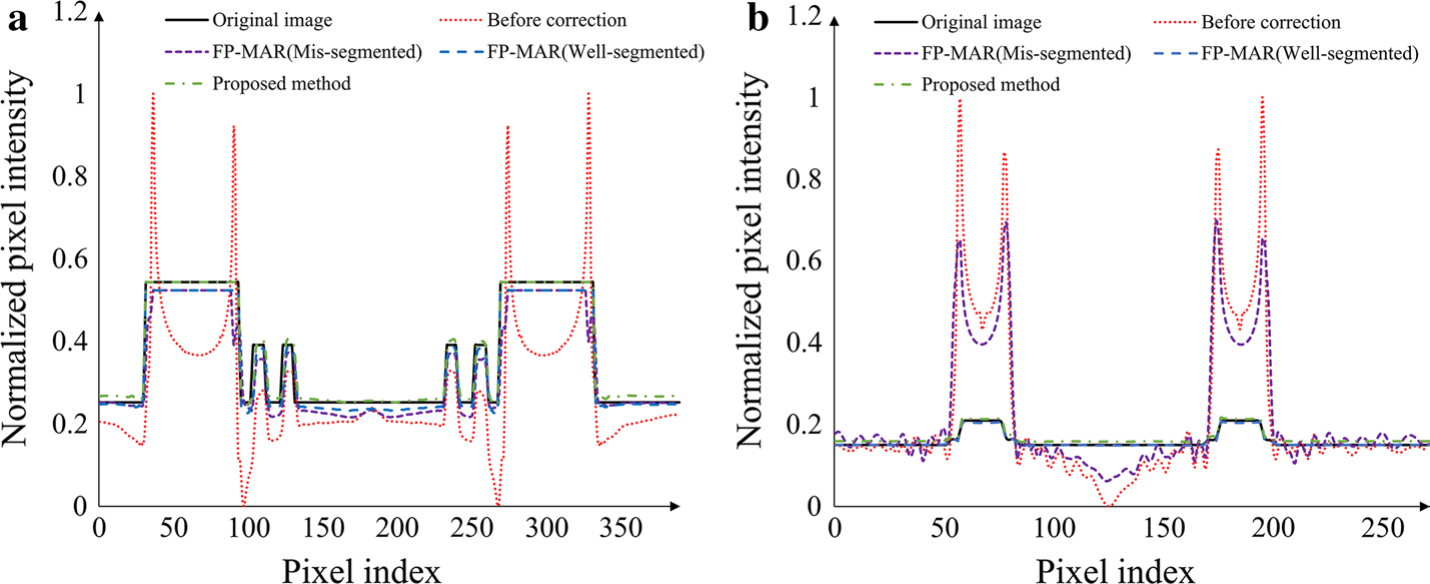
**a** The pixel intensity profiles along the line shown in Fig. 1a for the bilateral prostheses phantom. **b** The pixel intensity profiles along the line shown in Fig. 1c for the dental phantom

Figure 9a, b show the pixel intensity profiles along the solid lines shown in Fig. 1a, c, respectively. Before the correction, the profiles show high overshoots and undershoots around the metal region in both the cases. The FP-MAR method shows a profile similar to the proposed method when the optimal threshold was used, but it shows residual overshoots and undershoots when the lower threshold was used. Table 1 summarizes the similarity measures at the ROI A and B shown in Fig. 1. In both the simulation studies, SSIM is low when the images are not corrected or corrected by the FP-MAR method with some wrong segmentation. SSIM of the proposed method is very close to the ones of the FP-MAR method with optimal segmentation in both phantom images.

**Table 1:** Structural similarity index (SSIM) values at ROI A and B

	ROI A	ROI B
Bilateral prostheses phantom		
Uncorrected	0.832	0.715
FP-MAR (mis-segmented)	0.890	0.913
FP-MAR (well-segmeted)	0.995	0.993
Proposed method	0.995	0.994
Dental phantom		
Uncorrected	0.277	0.708
FP-MAR (mis-segmented)	0.398	0.613
FP-MAR (well-segmeted)	0.988	0.991
Proposed method	0.989	0.992

**Fig. 10:**
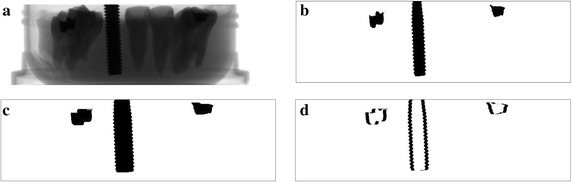
An example of the metal segmentation for the dental phantom shown in Fig. 6a. **a** A projection image of the dental phantom. **b** The metal regions segmented by the global thresholding. **c** The metal regions segmented by the proposed method. **d** The difference between **b** and **c**

Figure 10 shows the metal segmentation results of the dental phantom shown in Fig. 6a. Figure 10a is one of the projection images acquired at the CT scan in which the metal implant is clearly seen. Two dental fillings are also seen less conspicuous than the metal implant. Figure 10b, c are the segmentation results made by the global thresholding and the proposed method, respectively. The metal regions in Fig. 10c appear larger than those in Fig. 10b after elaborating with the adaptive local thresholding. Figure 10d is the difference between Fig. 10b, c.

We corrected the projection data of the two dental phantoms shown in Fig. 6, acquired by the micro-CT, by the proposed method and by the global thresholding for the FP-MAR method. We used two global thresholds for the FP-MAR method, one is the same as the one used for the proposed method and the other is large enough to extract all the metal regions despite some misclassifications of teeth as metallic objects. From the corrected projection data, we reconstructed 3D images using SART. With the aids of GPU-based parallelization, the computation time for reconstructing 3D images with a matrix size of 512 × 512 × 512 was 12 min from the projection images with a matrix size of 1120 × 1120 and 720 views.

**Fig. 11:**
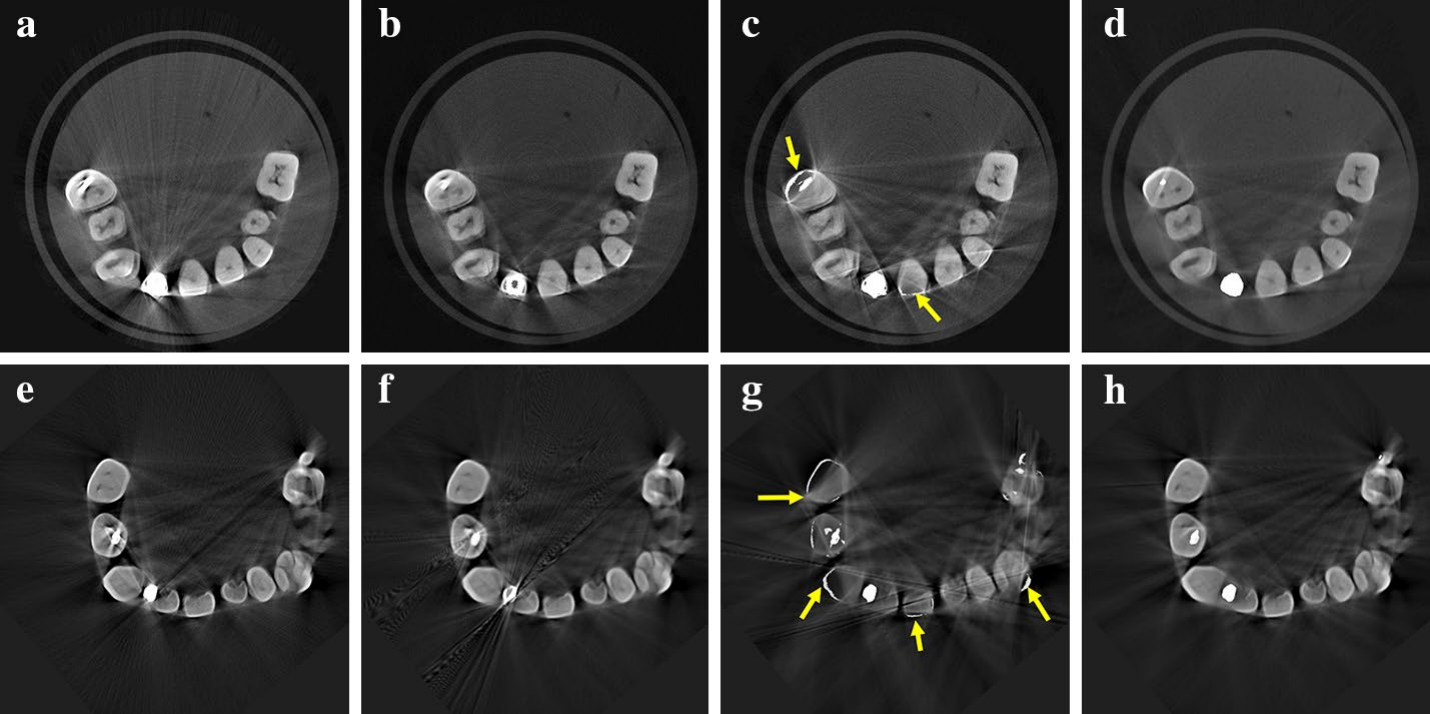
The upper and lower rows are the images of the dental phantoms shown in Fig. 6a, b, respectively. **a**, **e** The images reconstructed without metal artifact correction. **b**, **f** The images reconstructed by the FP-MAR method with a global threshold of 0.07 (partial segmentation of the metallic objects). **c**, **g** The images reconstructed by the FP-MAR method with a global threshold of 0.1 (full segmentation of the metallic objects). **d**, **h** The images reconstructed by the proposed method with a global threshold of 0.07 (*p* = 10, *c* = 0.1)

The upper and lower rows in Fig. 11 show the images of the dental phantoms shown in Fig. 6a, b, respectively. Figure 11a, e show the images of the dental phantoms without any metal artifact correction. Figure 11b, c show the images corrected by the FP-MAR method with global thresholds of 0.07 and 0.1, respectively, and Fig. 11d shows the image corrected by the proposed method with a global threshold of 0.07. Figure 11f, g show the images corrected by the FP-MAR method with global thresholds of 0.07 and 0.1, respectively, and Fig. 11h shows the image corrected by the proposed method with a global threshold of 0.07. With the lower threshold in Fig. 11b, f, metal artifacts still persist. With the higher threshold in Fig. 11c, g, some tooth regions, indicated by the arrows, are classified as metal regions causing contrast anomaly as well as residual streak artifacts. Figure 11d, h show less streak artifacts than the images obtained by the FP-MAR method without introducing any contrast anomaly.

## Discussion

Unlike the FP-MAR method, we applied the metal segmentation to the projection images in which metal artifacts do not appear, and we made streak-free images to generate the prior images. We used the prior images to make the replacement data at the metal trace on the projection images, thereby reducing the metal artifacts in the final image reconstruction. A simple global thresholding of the projection images may result in wrong metal segmentation, either smaller or bigger size than the original size, due to overlapping of other structures. However, adaptive local thresholding based on the pixel intensity statistics in and around the metal regions secured better metal segmentation than the global thresholding. The FP-MAR method showed residual metal artifacts when the global thresholding left some metal parts unsegmented. If the global threshold was set so as to segment all the metal parts in the FP-MAR method, streak artifacts could be reduced but with some contrast anomaly at the region of wrong segmentation. We observed that setting an optimal global threshold for the FP-MAR method in experimental dental images was not easy since the teeth caused x-ray attenuation not very different from the metallic objects. In the forward-projection-based method for metal artifact correction, generation of high-quality prior images is essential. Adaptive local thresholding is widely used in medical image processing such as bone segmentation in CT images [27, 28]. Owing to the adaptive local thresholding of the projection images in which streak artifacts are not present, we could generate higher quality prior images.

The proposed method would find some applications in dental CT when the patient has many metallic objects such as dental implants, crowns, and amalgam fillings. If there are too strong streak artifacts in dental CT images, segmenting the metallic objects without misclassifying the streak artifacts as metallic objects will not be easy. We need further studies to verify the proposed method with some clinical data. The dental phantoms we used have simple structures, but bony structures such as an alveolar bone, a maxillary bone and a mandibular bone are present around the teeth in a real human. The bony structures would make it challenging to segment metal regions on the projection images of a real human.

## Conclusions

In both the simulations and experimental studies with a micro-CT, the proposed method significantly reduced metal artifacts in dental CT images. The proposed method would have some advantages when the dental CT images have strong streak artifacts caused by multiple metallic objects.

## Abbreviations

CT: computed tomography
MAR: metal artifact reduction
FP-MAR: prior-based metal artifact reduction
ART: algebraic reconstruction technique
SART: simultaneous algebraic reconstruction technique
SSIM: structural similarity
ROI: regions of interest
SOD: source to object distance
SDD: source to detector distance

## References

[1] Glover GH, Pelc NJ (1981). An algorithm for the reduction of metal clip artifacts in CT reconstructions. Med Phys.

[2] Xu C, Verhaegen F, Laurendeau D, Enger SA, Beaulieu L (2011). An algorithm for efficient metal artifact reductions in permanent seed implants. Med Phys.

[3] Wei J, Chen L, Sandison GA, Liang Y, Xu LX (2004). X-ray CT high density artefact suppression in the presence of bones. Phys Med Biol.

[4] Zhao SY, Roberston DD, Wang G, Whiting B, Bae KT (2000). X-ray CT metal artifact reduction using wavelets: an application for imaging total hip prostheses. IEEE Trans Med Imaging.

[5] Zhang Y, Zhang L, Zhu XR, Lee AK, Chambers M, Dong L (2007). Reducing metal artifacts in cone-beam CT images by preprocessing projection data. Int J Radiat Oncol Biol Phys.

[6] Zhao S, Bae KT, Whiting B, Wang G (2002). A wavelet method for metal artifact reduction with multiple metallic objects in the field of view. J X-ray Sci Technol.

[7] Veldkamp WJH, Joemai RMS, van der Molen AJ, Geleijns J (2010). Development and validation of segmentation and interpolation techniques in sinograms for metal artifact suppression in CT. Med Phys.

[8] Wu J, Shih T, Chang SJ, Huang TC, Sun JY, Wu TH (2011). Metal artifact reduction algorithm based on model images and spatial information. Nucl Instrum Methods Phys Res A.

[9] Prell D, Kyriakou Y, Beister M, Kalender WA (2009). A novel forward projection-based metal artifact reduction method for flat-detector computed tomography. Phys Med Biol.

[10] Bal M, Spies L (2006). Metal artifact reduction in CT using tissue-class modeling and adaptive prefiltering. Med Phys.

[11] Meyer E, Raupach R, Lell M, Schmidt B, Kachelriess M (2010). Normalized metal artifact reduction (NMAR) in computed tomography. Med Phys.

[12] Wang J, Wang S, Chen Y, Wu J, Coatrieux JL, Luo L (2013). Metal artifact reduction in CT using fusion based prior image. Med Phys.

[13] Pua R, Wi S, Park M, Lee JR, Cho S (2016). An image-based reduction of metal artifacts in computed tomography. J Comput Assist Tomogr.

[14] Olive CS, Kaus MR, Pekar V, Eck K, Spies L (2004). Segmentation aided adaptive filtering for metal artifact reduction in radio-therapeutic CT images. Proc SPIE.

[15] Wang G, Vannier MW, Cheng PC (1999). Iterative x-ray cone-beam tomography for metal artifact reduction and local region reconstruction. Microsc Microanal.

[16] Wang G, Snyder DL, O’Sullivan JA, Vannier MW (1996). Iterative deblurring for CT metal artifact reduction. IEEE Trans Med Imaging.

[17] Zhang X, Wang J, Xing L (2011). Metal artifact reduction in x-ray computed tomography (CT) by constrained optimization. Med Phys.

[18] Snyder DL, O’Sullivan JA, Murphy RJ, Politte DG, Whiting BR, Williamson JF (2006). Image reconstruction for transmission tomography when projection data are incomplete. Phys Med Biol.

[19] De Man B, Nuyts J, Dupont P, Marchal G, Suetens P (2000). Reduction of metal streak artifacts in X-ray computed tomography using a transmission maximum a posteriori algorithm. IEEE Trans Nucl Sci.

[20] Sidkey EY, Pan XC (2008). Image reconstruction in circular cone-beam computed tomography by constrained total-variation minimization. Phys Med Biol.

[21] Yu HY, Wang G (2009). Compressed sensing based interior tomography. Phys Med Biol.

[22] Zhang Y, Yan H, Jia X, Yang J, Jiang SB, Mou X (2013). A hybrid metal artifact reduction algorithm for x-ray CT. Med Phys.

[23] Xia D, Roeske JC, Yu L, Pelizzari CA, Mundt AJ, Pan X (2005). A hybrid approach to reducing computed tomography metal artifacts in intracavitary brachytherapy. Brachytherapy.

[24] Park HS, Hwang D, Seo JK (2016). Metal artifact reduction for polychromatic x-ray CT based on a beam-hardening corrector. IEEE Trans Med Imaging.

[25] Wang Z, Bovik AC, Sheikh HR, Simoncelli EP (2004). Image quality assessment: from error visibility to structural similarity. IEEE Trans Image Processing..

[26] https://imagej.nih.gov/ij/plugins/ssim-index.html.

[27] Zhang J, Yan CH, Chui CK, Ong SH (2010). Fast segmentation of bone in CT images using 3D adaptive thresholding. Comput Biol Med.

[28] Wang LI, Greenspan M (2006). Validation of bone segmentation and improved 3-D registration using contour coherency in CT data. IEEE Trans Med Imaging.

